# Liposomal phytohemagglutinin: In vivo T‐cell activator as a novel pan‐cancer immunotherapy

**DOI:** 10.1111/jcmm.16885

**Published:** 2022-01-11

**Authors:** Kinan Alhallak, Jennifer Sun, Barbara Muz, Amanda Jeske, Julie O’Neal, Julie K. Ritchey, Samuel Achilefu, John F. DiPersio, Abdel Kareem Azab

**Affiliations:** ^1^ Department of Radiation Oncology Washington University School of Medicine in St. Louis St. Louis Missouri USA; ^2^ Department of Biomedical Engineering Washington University in St. Louis St. Louis Missouri USA; ^3^ Department of Medicine Washington University School of Medicine in St. Louis St. Louis Missouri USA; ^4^ Department of Radiology Washington University School of Medicine in St. Louis St. Louis Missouri USA

**Keywords:** immunotherapy, nanoparticles, T‐cell activation

## Abstract

Immunotherapy is an attractive approach for treating cancer. T‐cell engagers (TCEs) are a type of immunotherapy that are highly efficacious; however, they are challenged by weak T‐cell activation and short persistence. Therefore, alternative solutions to induce greater activation and persistence of T cells during TCE immunotherapy is needed. Methods to activate T cells include the use of lectins, such as phytohemagglutinin (PHA). PHA has not been used to activate T cells in vivo, for immunotherapy, due to its biological instability and toxicity. An approach to overcome the limitations of PHA while also preserving its function is needed. In this study, we report a liposomal PHA which increased PHA stability, reduced toxicity and performed as an immunotherapeutic that is able to activate T cells for the use in future cancer immunotherapies to circumvent current obstacles in immunosuppression and T‐cell exhaustion.

## 1 INTRODUCTION

T‐cell‐based immunotherapy is a promising approach for manipulating T cells to combat cancer. Multiple T‐cell immunotherapies have been rendered successful with impressive clinical outcomes. Chimeric antigen receptor (CAR)‐T cells are T cells extracted from the patient, genetically engineered to targets a tumour antigen, activated and expanded, and injected back into the patient. CAR‐T cells are highly activated and eliminate target cells in one single injection; however, they are challenged by cost, toxicity, complex production and antigen‐less tumour escape.^1^ Bispecific T‐cell engagers (TCEs) are composed of two single‐chain variable fragments connected by a protein linker, which bind CD3 on T cells and a tumour antigen on either ends. While TCEs demonstrate high efficacy and circumvents T‐cell engineering, they are challenged by particularly short half‐life, antigen‐less tumour escape (similar to CAR‐Ts) and weak T‐cell activation and persistence compared to CAR‐Ts.^2^

To overcome the lower T‐cell activation following treatment with TCEs, we aimed to exogenously activate T cells in vivo. Commonly used compounds to improve activation of T cells ex vivo include antibody‐conjugated beads, as well as small molecules and lectins, such as phorbol 12‐myristate 13‐acetate (PMA), ionomycin, concanavalin A and phytohemagglutinin (PHA).^3^, ^4^ Beads cannot be used in vivo, since they will be mainly accumulating in filtrating organs, such as the liver, and missing the target tumour tissue. PMA and ionomycin stimulate T cells by activating protein kinase C^5^; however, their use is limited by their carcinogenic potential.^6^ Concanavalin A and PHA, both lectins, stimulate T cells by binding to glycoproteins on the T‐cell receptor.^5^, ^7^, ^8^ PHA is more potent compared to concanavalin A, yet PHA has not been used to activate T cells in vivo due to its biological instability, short bioavailability profile and toxicity (ie agglutination of red and white blood cells) leading to death.^8^, ^9^, ^10^

Herein, we propose an alternative solution for the lower activation of TCE immunotherapy. In order to take full advantage of PHA as an immune activator, while also preserving its function, we encapsulated PHA in a liposome to increase its stability, reduce toxicity and create an immunotherapeutic circumventing current obstacles in immunosuppression and T‐cell exhaustion.

## 2 RESULTS AND DISCUSSION

**FIGURE 1:**
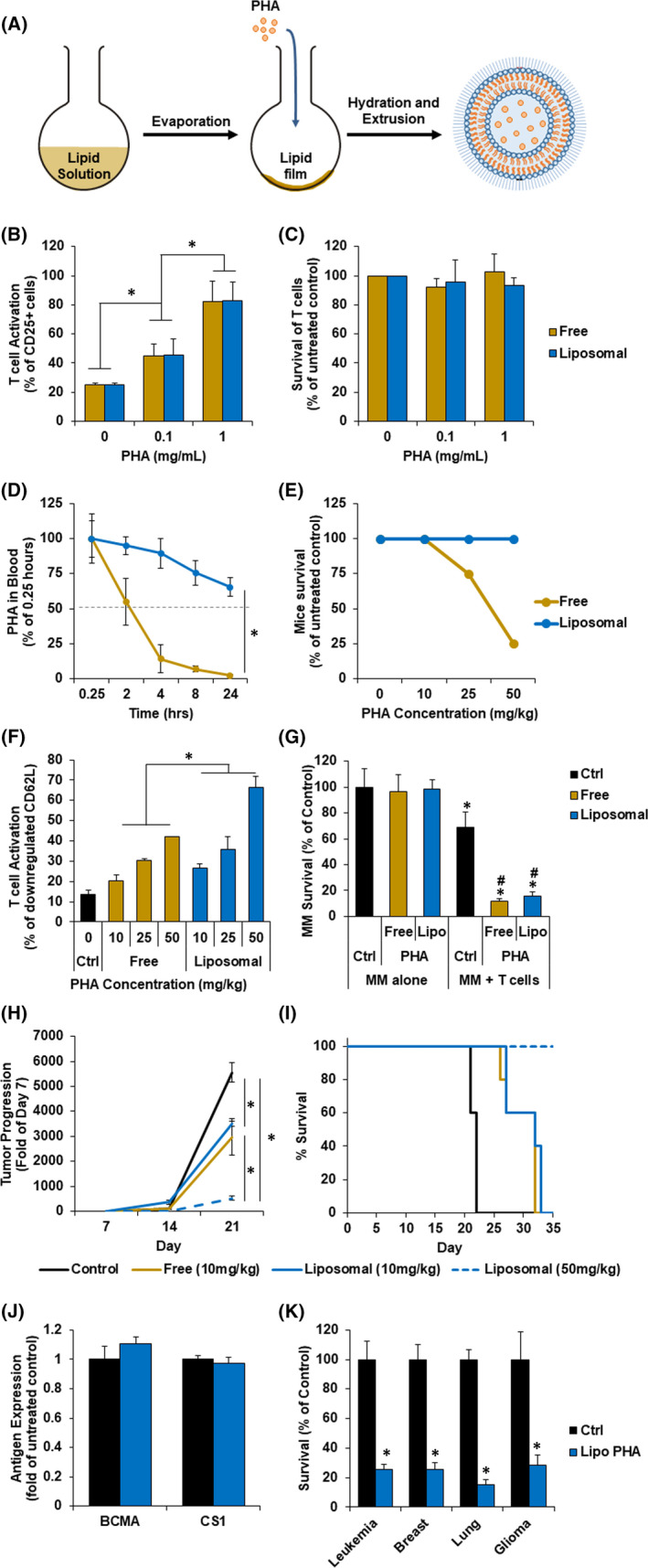
Liposomal phytohemagglutinin (PHA) has similar T‐cell activation, improves pharmacokinetic profile and reduces toxicity, compared to free PHA. (A) Schematic for preparation of liposomal PHA using the thin film hydration method. Liposomes were composed with three lipids: DPPC, cholesterol and 16:0 PEG2000 PE at a molar ratio of 60:30:10. These lipids were dissolved in chloroform, evaporated to form a thin lipid film, hydrated with PHA suspended in PBS and extruded through 100 nm polycarbonate membranes to produce uniform unilaminar particles. (B) Activation of T cells at increasing concentrations of free or liposomal PHA in vitro. Peripheral blood mononuclear cells (PBMCs) were incubated with 0, 0.1 or 1 mg/ml of free or liposomal PHA for 24 h, then stained with anti‐CD3 FITC and anti‐CD25 APC antibodies and analysed via flow cytometry. T‐cell activation is represented as % of CD25+ among CD3+ T cells. Statistical significance calculated by Student's *t* test is indicated by * (*p *< 0.05). (C) Survival of T cells at increasing concentrations of free or liposomal PHA. PBMCs were incubated with 0, 0.1 or 1 mg/ml free or liposomal PHA for 24 h, stained with anti‐CD3 FITC antibodies and analysed via flow cytometry. T‐cell survival was analysed as count of CD3+ cells against counting beads and represented as % of respective no treatment control. (D) Pharmacokinetic profile of free and liposomal PHA. PHA was labelled with a fluorescent dye Alexa Fluor 647 (AF647). In brief, 25 mg of PHA was dissolved in 500 µl of 0.1 M sodium carbonate, excess of AF647 was added, stirred for one hour at room temperature, and unbound AF647 was removed using dialysis. Immunocompetent C57BL/6 mice (Charles River Laboratories, strain 027, female, 52 days old) were injected intravenously with free or liposomal AF647‐PHA (10 mg/kg; *n* = 3 per group), and blood serum was analysed at 0.25, 2, 4, 8 and 24 h using a fluorescent plate reader (Ex/Em = 644/665). Statistical significance between two formulation calculated by 2‐way anova is indicated by * (*p *< 0.05). (E) Mice survival at increasing concentrations of free or liposomal PHA. C57BL/6 mice (*n* = 4 per concentration, per condition) were injected with increasing doses (0, 10, 25 and 50 mg/kg) of free or liposomal PHA and closely monitored for survival for three days. (F) Activation of T cells at increasing concentrations of free or liposomal PHA in vivo. C57BL/6 mice (*n* = 4 per concentration, per condition) were injected intravenously with 10, 25 or 50 mg/kg of free or liposomal PHA. Three days following injection, blood was extracted, and T‐cell activation was measured as the downregulation of CD62L expression on CD3+ T cells by flow cytometry. Activated T cells were presented as % CD3 T cells minus the T cells with high CD62L expression. Statistical significance between free and liposomal PHA calculated by 2‐way anova is indicated by * (*p *< 0.05). (G) Multiple myeloma (MM) survival at increasing concentrations of free or liposomal PHA. Fluorescently labelled (DiO) MM cell line OPM2 was cultured with or without PBMC isolated T cells and was treated with 1 mg/ml of free or liposomal PHA for 24 h, and the survival of MM cells was analysed using flow cytometry as count of OPM2 cells normalized against counting beads. Statistical significance between MM versus MM + T cells groups is indicated by * (*p *< 0.05); in MM + T cells condition, statistical significance of free or liposomal PHA compared to control is indicated by # (*p *< 0.05). (H) Tumour progression in MM‐bearing mice upon free of liposomal PHA treatment. Luciferase‐expressing mice myeloma 5TGM1 cells (1 × 10^6^ cells/mouse) were injected intravenously into 20 C57BL/KaLwRij mice. One week post‐inoculation, mice were randomly divided into 4 groups (*n* = 5) and treated intravenously with (A) vehicle control; (B) free PHA (10 mg/kg); (C) liposomal PHA (10 mg/kg); and (D) liposomal PHA (50 mg/kg). (I) Survival for MM‐bearing mice upon free of liposomal PHA treatment. Treatment groups are (A) vehicle control; (B) free PHA (10 mg/kg); (C) liposomal PHA (10 mg/kg); and (D) liposomal PHA (50 mg/kg). (J) Effect of liposomal PHA on MM antigen expression. Fluorescently labelled (DiO) MM cell line OPM2 was co‐cultured with T cells and treated with or without 1 mg/ml of liposomal PHA for 24 h. Cells were stained with anti‐BCMA or anti‐CS1 APC antibodies and analysed with flow cytometry. (K) Effect of liposomal PHA on various cancer survival. Leukaemia (THP‐1), breast cancer (MDA‐MB‐231), lung cancer (A549), and glioma (D54) cells were fluorescently labelled (DiO) and co‐cultured with PBMCs. Co‐cultures were treated with or without 1 mg/ml liposomal 24 h. The survival of cancer cells was analysed by flow cytometry, determined by cell count normalized to counting beads, and represented as % of control. Statistical significance is indicated by * (*p *< 0.05)

Liposomal formulations of PHA were prepared using the thin‐film hydration method^11^, ^12^ in which PHA is encapsulated in the core of the liposome particles (Figure 1A). Next, we investigated the ability of the PHA‐loaded liposomes to activate T cells compared to free PHA. We found that the T‐cell activation marker CD25 increased in expression correlating with PHA concentration, regardless of its formulation, and at 1 mg/ml, around 90% of T cells had an increase in CD25 expression (Figure 1B). We then examined the effect of PHA on T‐cell survival, as a marker for toxicity. No change in T‐cell survival was observed at 24 h for either formulations of PHA (Figure 1C). These results demonstrate that the liposomal formulation maintained the desired effect of T‐cell activation without changing the toxicity profile in vitro.

The main limitation of PHA is its low bioavailability and toxicity in vivo. Therefore, we investigated the effect of the liposomal formulation on PHA’s pharmacokinetic profile and toxicity in vivo. As expected, free PHA was degraded rapidly and had an elimination half‐life of around two hours, in mice. On the contrary, liposomal PHA showed an increased biological stability and pharmacokinetics in vivo, resulting in a longer half‐life of about 50 h (Figure 1D).

We then investigated the effect of the liposomal formulation on the toxicity of PHA in vivo. It has been previously reported that PHA induced death of animals within few hours after injection.^8^ Mice treated with free PHA showed decline in survival with increasing PHA concentration: 10 mg/kg did not show toxicity, 25 mg/kg induced death in 1 of 4 animals, and 50 mg/kg induced death in 3 of 4 animals, over 50% of the group, where the dose escalation was stopped. On the contrary, liposomal formulation induced no death of any of the treated animals at any of the doses, massively improving PHA’s toxicity profile (Figure 1E).

We next tested if liposomal PHA maintained efficacy in activating T cells in vivo. We saw that free and liposomal PHA both induced T‐cell activation in a dose‐dependent manner; however, liposomal PHA showed significantly higher T‐cell activation compared to free PHA at all doses (Figure 1F). These results demonstrate that the liposomal PHA not only overcame the stability and toxicity limitations of PHA in vivo, but also maintained—and even improved—the efficacy of PHA in T‐cell activation, most likely due to the improved bioavailability in vivo.

Multiple myeloma (MM) is a cancer of plasma cells and represent the second most common type of haematologic cancer. Despite recent advances in therapeutic options, the disease is still incurable and novel therapy options are direly needed.^11^, ^12^ We investigated the effect of free or liposomal PHA on the survival of MM cells, directly or indirectly as an immune activator of T cells. Neither free nor liposomal PHA demonstrated direct killing on MM cells in absence of T cells. However, when co‐cultured with T cells, both free and liposomal abolished MM survival in vitro (Figure 1G). These results emphasize that the robust effect of PHA is mediated by T‐cell activation.

To demonstrate the efficacy of liposomal PHA as an immune activator for the treatment of cancer, we tested its efficacy in an immunocompetent MM mouse model using C57BL/KaLwRij mice. Mice were treated weekly with: (a) vehicle control; (b) free PHA (10 mg/kg; maximal tolerated dose); (c) liposomal PHA (10 mg/kg; comparable to free PHA); and (d) liposomal PHA (50 mg/ml; highest tested dose which did not show toxicity in vivo). No free PHA was used at the high concentration since it induced immediate death of more than 50% of the animals. 10 mg/kg free and liposomal PHA performed similarly and delayed tumour progression compared with control. However, the 50 mg/kg of liposomal PHA significantly abrogated the tumour progression (Figure 1H). The control cohort died at day 22, 10 mg/kg free and liposomal PHA had 60% of mice survived past day 31; whereas 50 mg/kg liposomal PHA‐treated mice had 100% survival up to day 35 (Figure 1I).

Moreover, typical cancer immunotherapies such as CAR‐T cells and TCEs target a specific marker on the tumour cell for elimination; however, this leads to antigen‐less clones and relapse.^13^, ^14^ BCMA and CS1 are both popular MM targets for immunotherapy, and previous therapies targeting each of these antigens were shown to induce a decrease in the expression and induce antigen‐less tumour escape in MM.^15^ We therefore tested the effect of the liposomal PHA on the expression of these antigens in MM cells. We observed that liposomal PHA did not reduce the expression of BCMA or CS1 expression on MM cells (Figure 1J). This is because the liposomal PHA does not depend on a specific antigen.

Finally, and due to the fact that the liposomal PHA activates T cells generically regardless of the tumour target, we exploited the use of liposomal PHA for the treatment of a variety of cancers such as leukaemia, breast cancer, lung cancer, and glioma. Following 24 h treatment of liposomal PHA in the presence of T cells in vitro, we observe a significant killing of all cell types by T cells in the liposomal PHA group compared to untreated control (Figure 1K).

In summary, liposomal PHA improved T‐cell activation in vivo and presented superior pharmacokinetic and safety profiles compared to free PHA, resulting in significant delayed tumour progression and prolonged survival of cancer‐bearing mice in vivo. Importantly, liposomal PHA does not include targeting to a particular tumour antigen and is effective against multiple cancer types, including haematologic and solid tumours, without inducing antigen‐less tumour escape. Liposomal PHA represents a novel therapy that can be off‐the shelf, highly effective, T‐cell immunotherapy without complex manufacturing and safety concerns. Further studies are needed to investigate the use of PHA liposomes in combination with other T‐cell therapies, and traditional chemo‐ and biological therapies.

## CONFLICT OF INTEREST

A.K.A and K.A. have filed a patent with regards to the technology described in this study. A.K.A is the founder and owner of Cellatrix LLC and Targeted Therapeutics LLC; however, these entities had no contribution to the current study. Other authors state no conflicts of interest.

## AUTHOR CONTRIBUTIONS

**Kinan Alhallak:** Formal analysis (lead); Investigation (lead); Methodology (lead); Visualization (lead); Writing‐original draft (lead). **Jennifer Sun:** Formal analysis (equal); Investigation (equal); Writing‐review & editing (equal). **Barbara Muz:** Formal analysis (equal); Investigation (equal); Writing‐review & editing (equal). **Amanda Jeske:** Formal analysis (supporting); Investigation (supporting); Writing‐review & editing (supporting). **Julie O’Neal:** Formal analysis (supporting); Investigation (supporting). **Julie K. Ritchey:** Formal analysis (supporting); Investigation (supporting). **Samuel Achilefu:** Methodology (equal); Resources (equal). **John F. DiPersio:** Methodology (equal); Resources (equal). **Abdel Kareem Azab:** Conceptualization (equal); Formal analysis (equal); Methodology (equal); Visualization (equal); Writing‐review & editing (equal).

## Data Availability

All data generated are available upon request.
